# Prolonged improvement of childhood onset systemic lupus erythematosus following systematic administration of rituximab and cyclophosphamide

**DOI:** 10.1186/1546-0096-12-3

**Published:** 2014-01-14

**Authors:** Thomas JA Lehman, Chahait Singh, Anusha Ramanathan, Risa Alperin, Alexa Adams, Laura Barinstein, Nandini Moorthy

**Affiliations:** 1 Hospital for Special Surgery, Weil Cornell Medical College, New York, NY, USA; 2 Rutgers University, Robert Wood Johnson Medical School, New Brunswick, New Brunswick, NJ, USA

## Abstract

**Background:**

Although the combination of cyclophosphamide and rituximab has been utilized in case reports, there are no previous reports of the long term outcome of SLE treated systematically with this regimen. We report a pilot study to evaluate the efficacy of a systematically administered course of rituximab and cyclophosphamide over an eighteen month period to provide sustained improvement in childhood onset systemic lupus erythematosus (SLE).

**Findings:**

Twelve patients with childhood onset lupus nephritis or corticosteroid resistant SLE received systematic treatment with a combination of rituximab (750 mg/M^2^ up to 1 gram) and cyclophosphamide (750 mg/M^2^: no patient exceeded 1.8 M^2^). Two administrations of rituximab and cyclophosphamide, two weeks apart, were administered at the start of study, six months later, and eighteen months later. Clinical data were collected and analyzed after sixty months of follow up. There was sustained improvement in all clinical parameters with a dramatic reduction in both mean SLEDAI score (10.1 to 1 at one year and 0 at five years p<0.005) and mean daily prednisone dosage (29.7 mg/day to 12.7 by one year and 7.0 mg/day at five years p<0.005), with sustained improvement in mean C3 (55.5 mg/ml to 113 at one year and 107.5 at five years p<0.001) which was maintained through sixty months of follow up. Serum immunoglobulin levels were transiently depressed but mean values were within the normal range for both IgG and IgM at one and five years. Few complications were observed (two episodes of febrile neutropenia during the first year of treatment were the only serious adverse events) and patients routinely reported sustained wellbeing.

**Conclusions:**

This pilot study demonstrates that a systematically administered course of rituximab and cyclophosphamide over an eighteen month period provided sustained relief for patients with childhood onset SLE which was maintained over a sixty month period, while minimizing the need for corticosteroids, without excessive toxicity.

## Findings

This study demonstrates the long term safety and efficacy of a limited course of concurrent rituximab and cyclophosphamide administered in a systematic fashion to twelve patients with five years of follow-up. This therapy allowed both substantial reduction in the total dosage of cyclophosphamide and eliminated the need for continued oral therapy with corticosteroids in doses above 0.25 mg/kg/day, while providing sustained clinical improvement. The short term results of this therapy have previously been reported in abstract form.

The care of patients with childhood onset SLE is complicated by frequent noncompliance with the prescribed medication regimen. This results in part from the adverse effects of corticosteroids on appearance, but noncompliance among lupus patients is common with many medications [1]. Noncompliance has been documented with hydroxychloroquine which requires only a single daily dose with rare side effects and is common with mycophenolate mofetil which requires multiple daily doses associated with gastrointestinal side effects [2,3]. Noncompliance is strongly associated with an increased frequency of disease flares, increased morbidity, and poor outcome [4].

Multiple approaches to the problem of noncompliance have been proposed. These include educational programs, electronic monitoring, and automated medication reminders [5-7]. However, the optimal solution is a regimen that both maximizes the physician's ability to monitor compliance and minimizes the patient's need for continued therapy. In the past, intravenous cyclophosphamide has been a standard regimen for the treatment of life-threatening active childhood onset SLE [8-11]. Compliance with intravenous cyclophosphamide is easily monitored, but patients and physicians remain concerned about the long term side effects [12,13]. The risks of infection, sterility, and malignancy, and other toxicities lead to reluctance to accept this therapy.

Efforts to develop alternative regimens with similar or better efficacy and safety than repeated intravenous cyclophosphamide administration have focused on mycophenolate mofetil [14] and biologic agents such as rituximab. Although intravenous rituximab has been beneficial in many case reports, it has lacked efficacy in controlled trials [15,16]. While rituximab targets only CD20 positive B cells, cyclophosphamide is an alkylating agent which targets all rapidly dividing cell types [17].

## Methods

Patients with childhood onset SLE complicated by active diffuse proliferative glomerulonephritis ( DPGN), or who failed to attain adequate disease control to allow appropriate reduction in the corticosteroid dosage during a minimum three month trial were offered the opportunity to participate. Appropriate reduction in corticosteroid therapy was defined as a reduction in the daily dose of prednisone or equivalent to ≤ 0.25 mg/kg/day. Additional medications such as hydroxychloroquine or angiotensin inhibitors were added or withdrawn at the discretion of the attending physician. Prior therapy varied from case to case and in some cases included mycophenolate mofetil or cyclophosphamide without adequate response as defined by disease control with less than 0.25 mg/kg/day of prednisone or equivalent. In each case the expected risks and benefits and the novel nature of the regimen were explained and informed consent was obtained. This report is limited to 12 patients who have completed five years of follow-up.

**Figure 1:**
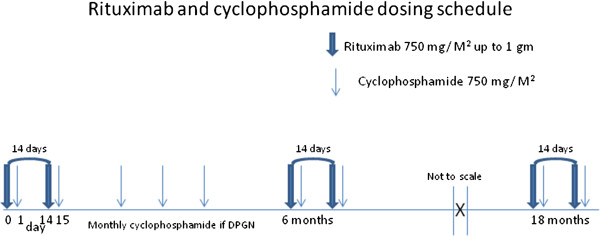
Rituximab and cyclophosphamide dosing schedule.

Rituximab and cyclophosphamide were administered as inpatient intravenous infusions in all cases. Over eighteen months each patient received a course of therapy consisting of six infusions of rituximab 750 mg/M^2^ (up to a maximum dose of 1 gram per infusion), followed twenty-four hours later by cyclophosphamide at 750 mg/M^2^. The infusions were given in three sets of two. Thus, a patient received rituximab on day 0, cyclophosphamide on day 1 and then rituximab on day 14 and cyclophosphamide on day 15 in each set. As illustrated in Figure 1, each patient received a set of infusions at the start of therapy, at six months, and at eighteen months. Patients with active DPGN received additional infusions of cyclophosphamide 750 mg/M^2^ at six, ten, and fourteen weeks after the start of therapy. Pertinent laboratory data, prednisone dosage, and SLEDAI were recorded at the time of each initial admission for a set of infusions and at six month intervals after the set of infusions was completed. Five year results are presented here, long term follow up of these patients and data collection continues.

## Results

Twelve patients have completed more than sixty months of follow up. Nine were female and three male, with an average age at diagnosis of 12.5 years and at start of therapy of 16 years (range 10–28 years). Five were Caucasian, four were Hispanic, three were Asian. Eight patients had active DPGN; the other four did not, but were unable to tolerate appropriate reduction in their corticosteroid dosage without SLE flare manifested by hypocomplementemia, elevated ESR and systemic symptoms.

Combined therapy with rituximab and cyclophosphmaide resulted in significant reduction in mean prednisone dosage, erythrocyte sedimentation rate, and SLEDAI at one year which persisted through year five [see Table 1]. These changes were accompanied by significant rises in mean C3, serum albumin, and hemoglobin. See Table 1. Mean serum albumin rose from 3.37 gms/dl at the beginning of treatment to 4.08 gms/dl at month 12 (p < 0.025) and remained improved at 4.26 gms/dl at month 60 (P < 0.025 vs month 0). Mean serum creatinine remained stable in the normal range throughout. CD19 levels returned to normal in all patients by one year (six months following the second set of rituxan and cyclophosphamide treatments) and were again normal at five years of follow up. Mean serum IgG level at the initiation of treatment was 1273 gms/dl at month 12 it was 944 gms/dl and at month 60 it was 969 gms/dl. These differences were not statistically significant. IgM levels varied widely, but the levels were reduced with a mean at one year of follow up of 44.8 mg/dl and a mean at five years of 51.8 mg/dl. Six patients remained between 20 and 40 mg/dl at one year, but none were lower than 20 mg/dl. At five years the IgM of five patients remained below 40 mg/dl and one was below 20 mg/dl. Although individual patients transiently had IgM levels below 5 mg/dl during the period of follow up, there were no serious infections.

**Table 1:** Sustained improvement at 12 and 60 months

	**Month 0**	**Month 12**	**Month 60**
Prednisone (mg/day)	29.7 ± 24^ṩ^	12.7 ± 4.1^#^	7.0 ± 2.5*
C3 (mg/ml)	55.5 ± 27	113 ± 32**	107.5 ± 36**
ESR (mm/hr)	42.3 ± 27	17.8 ± 22*	11.8 ± 12**
Hb (gms/dl)	11.3 ± 1.8	12.1 ± 1.7^#^	13.2 ± 1.4^#^
SLEDAI	10.10 ± 5.9	1.0 ± 1.8*	0*
IgG (mgs/ml)	1272 ± 526	819 ± 200*	977 ± 505*
IgM (mgs/ml)	107 ± 52	45 ± 24*	51 ± 28^#^

^ṩ^All values represent mean ±1 Standard deviation.

#p<0.05 *p<0.005 **p<0.001.

Four of the twelve patients remain on 10 mgs of prednisone daily, the remainder less. None are Cushingoid and all patients remained well through five years of follow-up. All were ANA positive initially, but 6 of the 12 became and remain ANA negative and anti-DNA negative. No patient required mycophenolate mofetil after receiving rituximab and cyclophosphamide. No patient experienced a life threatening complication and there were no infusion reactions to rituximab. Two patients were hospitalized emergently because of febrile neutropenia. Both cases responded promptly to broad spectrum antibiotic therapy despite negative blood, urine, and sputum cultures. Neither of the febrile neutropenic patients was admitted to the pediatric ICU as they were not toxic appearing or hypotensive.

## Discussion

The regimen of systematically administered cyclophosphamide and rituximab described here minimized the need for daily corticosteroid therapy over a sustained five years of follow up. While compliance with the admissions for rituximab and cyclophosphamide was easily monitored and assured, only routine efforts were made to encourage compliance with the daily dosage of corticosteroids and hydroxychloroquine [After six years one patient developed recurrent hematuria and a decreased C3 level which resolved following one additional set (two doses two weeks apart) of rituximab and cyclophosphamide infusions and has not recurred in eighteen months of further follow-up. A second patient, who had become ANA negative became ANA positive and progressively developed additional antibodies including antibodies to Sm during the sixth year. This patient elected to be retreated with one set of rituximab and cyclophosphamide infusions following which she no longer has antibodies to Sm, but remains ANA positive].

Intravenous cyclophosphamide is a well established alkylating agent which has long been utilized for the treatment of childhood onset SLE [8-11]. Although it is recognized as a potent antimetabolite the precise mechanism of its efficacy in SLE is unknown [17]. While previous studies have demonstrated that the systematic administration of intravenous cyclophosphamide provides significant relief for patients with SLE, significant concern remains regarding the long term toxicity of cyclophosphamide [12,13].

Although there are many prior case reports of improvement following a single treatment with cyclophosphamide and rituximab [18], there is only one prior report of the risks and benefits of systematically administering this therapy which utilized a different regimen and has shorter follow-up [19]. Rituximab has been demonstrated to efficiently eliminate the circulating pool of CD20 positive B cells, but does not eliminate the entire CD20 positive population in the body [16]. While B cells likely play an important role in the manifestations of SLE, the failure of controlled trials utilizing rituximab alone indicates that CD20 positive B cells do not act alone in the pathogenesis of SLE [15].

There was a sustained improvement with the combination of rituximab and cyclosphosphamide despite a reduction in the total cyclophosphamide dosage from 17 gms/M^2^ in the protocol previously utilized [10,11] to 6.75 gms/M^2^ or less (a reduction of over 60%). All of our patients were stable on a low daily dose of prednisone which was not associated with cushingoid faces or other clinically evident adversity within one year of starting therapy.

Mycophenolate mofetil has been shown to be effective for lupus nephritis in a number of controlled trials [14]. This regimen has not been compared to mycophenolate mofetil. It is simply noted that satisfactory results with mycophenolate mofetil are dependent on compliance which is inhibited by gastrointestinal side effects [3]. The package insert notes that mycophenolate mofetil is to be administered twice daily despite digestive side effects reported in more than twenty percent of patients [20].

This remains only a small number of patients. Nonetheless, none of the twelve patients reported have developed significant corticosteroid related complications and none manifest a "cushingoid" appearance. All report feeling, "like I don't have lupus anymore." The maximum current dose of prednisone is 10 mgs/day. In contrast to patients receiving seventeen doses of cyclophosphamide at 1 gm/M^2^ and hence a total dose of 17 gms/M^2^ over three years, these patients received at most 6.75 gms/M^2^ of cyclophosphamide over eighteen months. Similar results have been obtained for the patients subsequently receiving this regimen, but the emphasis of this manuscript is the sustained response.

Although we have Black patients receiving this regimen, none have yet completed five years of follow-up. There is concern that disease severity and medication efficacy may vary between races in patients with SLE [21].

The major advantage of this regimen was the ability to rapidly reduce the dosage of corticosteroids without disease flare and prevent (or rapidly resolve) the development of Cushingoid faces and other complications of corticosteroid therapy. All patients were compliant with the need for hospital admissions at the specified intervals and any noncompliance would have been immediately evident. While patients admit to occasionally forgetting their current dosage of prednisone (in most cases 5 mg daily) they do not report any resistance to this low dosage. It is uncertain whether it in fact remains necessary [22].

## Competing interests

The authors declare they have no competing interests.

## Authors’ contributions

This study was designed and implemented by TL. CS was responsible for data collection and verification. AR RA AA LB and NM all participated in the original design of the study, care of the patients, and analysis of the data and it’s interpretation. All authors read and approved the final manuscript.
